# Supplementary material for: Kölliker–Fuse/Parabrachial Complex PACAP—Glutamate Pathway to the Extended Amygdala Couples Rapid Autonomic and Delayed Endocrine Responses to Acute Hypotension
Source: Int J Mol Sci. 2025 Nov 25;26(23):11405. doi: 10.3390/ijms262311405 (PMC12692419; doi:10.3390/ijms262311405)
Supplement: Supplementary file 1 [file ijms-26-11405-s001.zip › Supplementary Tables.pdf]

Supplemental information

Supplementary Table S1. Individual heart frequency values: 3 measurements at each time point.

	MBP	Saline 1	Saline 2	Saline 3	HDZ 1	HDZ 2	HDZ 3	HDZ 4	HDZ 5	HDZ 6
**-10 min**	Measure 1	388	381	398	369	415	399	366	411	398
	Measure 2	405	362	369	370	397	419	369	399	395
	Measure 3	381	366	365	370	399	422	374	401	411
	**Average**	391.3	369.6	377.3	369.7	403.7	413.3	369.7	403.7	401.3
**0**	Measure 1	378	410	387	399	401	384	401	390	399
	Measure 2	365	368	377	400	375	387	399	390	378
	Measure 3	369	366	396	393	388	379	392	387	376
	**Average**	370.6	381.3	386.7	397.3	388.0	383.3	397.3	389.0	383.3
**5 min**	Measure 1	396	372	357	414	444	459	416	437	459
	Measure 2	393.0	351.0	366.0	417	443	486	414	440	468
	Measure 3	398.0	348.0	345.0	402	424	469	415	434	478
	**Average**	395.7	357.0	356.0	411.0	437.0	471.3	415.0	437.0	468.3
**10 min**	Measure 1	394.0	369.0	378.0	409	412	422	415	420	***364***
	Measure 2	368.0	373.0	403.0	415	419	411	415	425	***346***
	Measure 3	365.0	365.0	380.0	376	419	412	409	426	***343***
	**Average**	375.7	369.0	387.0	400.0	416.6	415.0	413.0	423.7	***351****
**15 min**	Measure 1	373.0	345.0	347.0	432	401	448	440	402	458
	Measure 2	363.0	319.0	349.0	423	395	449	421	379	447
	Measure 3	364.0	335.0	349.0	420	361	467	414	376	459
	**Average**	366.7	333.0	348.3	425.0	385.7	454.7	425.0	385.7	454.7
**30 min**	Measure 1	388.0	329.0	388.0	391	444	388	371	444	340
	Measure 2	359.0	357.0	388.0	362	439	401	370	438	318
	Measure 3	361.0	346.0	391.0	362	448	396	373	449	317
	**Average**	369.3	344.0	389.0	371.7	443.7	395.0	371.3	443.7	325.0
**60 min**	Measure 1	383.0	327.0	419.0	398	423	415	398	407	378
	Measure 2	368.0	328.0	390.0	379	418	415	369	416	344
	Measure 3	363.0	346.0	388.0	375	440	415	370	410	349
	**Average**	371.3	333.7	399.0	384.0	427.0	415.0	379.0	411.0	357.0
**90 min**	Measure 1	389.0	361.0	358.0	432			409	373	410
	Measure 2	386.0	359.0	364.0	422			400	378	413
	Measure 3	423.0	359.0	380.0	421			409	400	439
	**Average**	399.3	359.8	367.3	425.0			406.0	383.7	420.7
**120 min**	Measure 1	356.0	401.0	379.0				401	410	378
	Measure 2	361.0	374.0	377.0				400	399	373
	Measure 3	365.0	369.0	374.0				373	402	374
	**Average**	360.7	381.3	376.7				391.3	403.7	375.0

Supplementary Table S2. Individual mean blood pressure values: 3 measurements at each time point.

	MBP	Saline 1	Saline 2	Saline 3	HDZ 1	HDZ 2	HDZ 3	HDZ 4	HDZ 5	HDZ 6
**-10 min**	Measure 1	129	123	111	115.6	110.9	114	130	118.1	120
	Measure 2	124	126.7	112	116.7	111.6	110	126	118.3	115
	Measure 3	123	126	104	116.1	112.8	109	125	115	114
	**Average**	**125.3**	**125.2**	**109**	**116.1**	**111.7**	**111**	**127**	**117.1**	**116.3**
**0**	Measure 1	130	119	100	120.5	110.1	115	120	111	105.8
	Measure 2	127	111.6	103.9	116.5	113.4	114	114	110	106.1
	Measure 3	127	115	103	118.5	107.9	114	115.5	110	108
	**Average**	**128**	**115.2**	**102.3**	**118.5**	**110.4**	**114**	**116.5**	**110**	**107.6**
**5 min**	Measure 1	118	116	113	83.5	88.3	79.7	69	86.6	98
	Measure 2	111.5	117.5	113	80.2	93	77.9	63	79	97.5
	Measure 3	115	116	113	80.2	92.5	83.1	63	77.9	98.5
	**Average**	**114.8**	**116.5**	**113**	**81.3**	**92.2**	**80.25**	**65**	**81.1**	**98**
**10 min**	Measure 1	130.6	109.5	106	87	62	73	52	58	100
	Measure 2	125.6	109	102	82.9	54	70	48	54	100
	Measure 3	126.8	109	110	77.9	54	70	48	53	91
	**Average**	**127.6**	**109**	**106**	**82.6**	**56.6**	**71**	**49**	**55**	**97**
**15 min**	Measure 1	130	111	112	91	69	69	60	62	108
	Measure 2	123	108	107.6	84	75	66	59	57	103
	Measure 3	123	109.9	109	84	73	66	59	58	101
	**Average**	**125.3**	**109.6**	**109.5**	**86.3**	**72.3**	**67**	**59**	**59**	**104**
**30 min**	Measure 1	118	102.8	98	91.1	68	78.4	99	89	111.9
	Measure 2	118	99.9	97	87.7	70	74.5	90	85	108.6
	Measure 3	118	99.6	96	86	70	73.9	90	84	108
	**Average**	**118**	**100.7**	**97**	**88.2**	**69.3**	**75.6**	**93**	**86**	**109.5**
**60 min**	Measure 1	129	101	103	86	90	82	88	95	98
	Measure 2	123	103.9	100	78	85	77	87	99.7	98
	Measure 3	126	105	100	82	84	78	80	106.6	101
	**Average**	**126**	**103.3**	**101**	**82**	**86.3**	**79**	**85**	**100.4**	**99**
**90 min**	Measure 1	113	103	120	121			94	90	96.5
	Measure 2	111.8	106	114	118			90	90	88.1
	Measure 3	112	106	114	118			89	93	91.5
	**Average**	**112.25**	**105**	**116**	**119**			**91**	**91**	**92**
**120 min**	Measure 1	119.8	116	113.5				100	97.1	101
	Measure 2	109.8	117	111				100	90	96
	Measure 3	111	109	111.5				97	90.5	96
	**Average**	**113.5**	**114**	**112**				**99**	**92.5**	**97.6**
