# Supplementary material for: Kölliker–Fuse/Parabrachial Complex PACAP—Glutamate Pathway to the Extended Amygdala Couples Rapid Autonomic and Delayed Endocrine Responses to Acute Hypotension
Source: Int J Mol Sci. 2025 Nov 25;26(23):11405. doi: 10.3390/ijms262311405 (PMC12692419; doi:10.3390/ijms262311405)
Supplement: Supplementary file 1 [file ijms-26-11405-s001.zip › Supplementary Figure.pdf]

Supplemental information (SI)

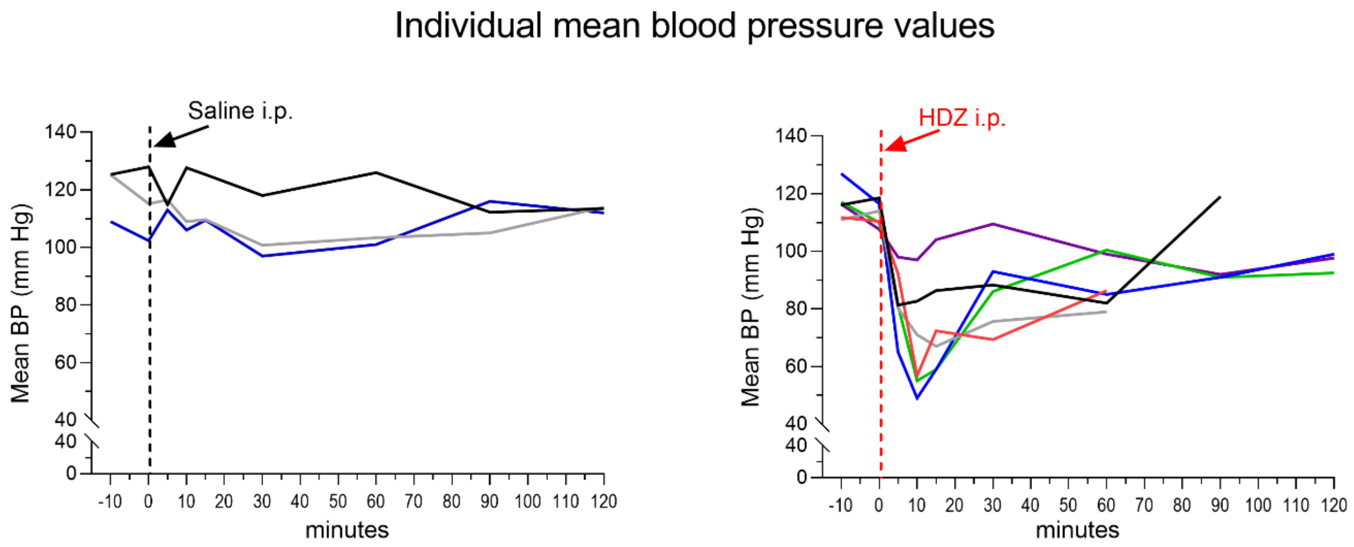

Supplementary Figure S1. Individual mean blood pressure values.
